# Randomized Controlled Trial Comparing the Effects of Preoperative Carbohydrate and Non-carbohydrate Loading on Gastric Emptying in Diabetic and Non-diabetic Patients Posted for Elective Surgery

**DOI:** 10.7759/cureus.49213

**Published:** 2023-11-21

**Authors:** Manoj Vishak, Balasubramaniam Gayathri, Gokulakrishnan Chandrasekhar, Swetha Ramani

**Affiliations:** 1 Anaesthesiology, SRM Medical College Hospital and Research Centre, Chennai, IND

**Keywords:** ultrasound (u/s), capillary blood glucose, diabetes, gastric volume, oral carbohydrate loading

## Abstract

Background

Preoperative fasting for six hours and accepting clear fluids till two hours of surgery is followed as a regular practice. Carbohydrate-rich fluids antagonize catabolism and are claimed to be tolerated better. This study aims to compare the effect of carbohydrate-rich drinks on gastric volume and blood sugar control in diabetic and non-diabetic patients undergoing elective surgery with plain water.

Methods

Two hundred forty patients aged 40 to 65 undergoing elective surgery under regional anesthesia were randomized into diabetic control, diabetic study, non-diabetic control, and non-diabetic study. Control groups were given 400 ml of plain water, while the study group received 50 grams of dextrose dissolved in 400 ml of water two hours prior to surgery. Gastric volume was evaluated using USG, and thirst and discomfort were assessed using the Likert scale. Perioperatively, blood sugar values were monitored and kept under control using insulin.

Results

Mean gastric volume (ml) in diabetic control (35.3±12.95 ml), diabetic study (31.2±11.75 ml), non-diabetic control (29±11.42 ml), and non-diabetic study (30.4±9.12 ml) showed no statistically significant difference (p>0.05). Capillary blood glucose (CBG) values two hours post fluid intake showed a significant increase in CBG levels in the diabetic study (183.2±28.67 mg/dl) compared to the diabetic control group (138.66±15.81 mg/dl). The values returned to baseline within six hours. Thirst and discomfort were significantly lower in the study group of diabetic and non-diabetic populations.

Conclusion

We conclude that carbohydrate loading does not affect gastric volume in diabetics and non-diabetics. However, the sugar values do increase which may warrant hourly checking and administration of insulin in diabetics.

## Introduction

Pulmonary aspiration resulting in respiratory failure has been documented in both emergency and elective surgical procedures, with an incidence rate of three cases per 100,000 procedures [1]. The Thai Anesthesia Incident Monitoring Study reported that 1.4% of total surgical incidents developed aspiration, while another report from Australia showed a 0.48% incidence [2,3]. It has been reported that passive regurgitation, as opposed to active vomiting, leads to aspiration in a substantial number of cases. Many of the patients who aspirated were found to have pre-existing symptoms of reflux or conditions that contribute to delayed gastric emptying, such as diabetes [4]. To reduce the risk of pulmonary aspiration, strict fasting guidelines have been advocated before surgery.

Typically, preoperative patients without any comorbidities have been found to have gastric fluid volumes ranging from 10 to 30 ml, while patients with comorbidities like gastroesophageal reflux disease (GERD) and diabetes mellitus may have volumes of up to 200 ml. While some studies have suggested that a threshold of 200 ml or more of gastric content can increase the risk of regurgitation and aspiration during induction, it's important to acknowledge that this threshold is not an absolute value and can vary among individuals [5]. At present, there is a lack of consensus regarding the appropriate fasting intervals for diabetic patients. The 2011 fasting guidelines from the European Society of Anesthesiology recommend that diabetic patients adhere to the same guidelines as healthy adults. However, the 2017 fasting guidelines from the American Society of Anesthesiologists (ASA) suggest that the standard eight-hour fasting period may need adjustments for patients with conditions that can impact gastric emptying or fluid volume [6].

Recent advancements in medical technology have enabled the reliable and rapid estimation of gastric volume in real-time using USG. Studies have investigated the impact of plain water or carbohydrate-free liquid intake on gastric volume in both diabetic and non-diabetic patients scheduled for surgery, and the findings recommend a safe limit of two hours [7]. However, it's important to note that there is a notable dearth of studies conducted in the Indian context that specifically address the issue of diabetic patients consuming carbohydrate-rich fluids prior to surgery. This lack of research is particularly evident when considering its implications for both gastric emptying and glycemic control. To bridge this knowledge gap and gain a deeper understanding of these crucial aspects, our study was meticulously designed. Its primary aim was to investigate whether significant differences exist in gastric volume between diabetic and non-diabetic patients following the consumption of carbohydrate-rich fluids. Furthermore, we sought to explore the potential benefits of carbohydrate-loaded drinks with regard to maintaining optimal glycemic control and enhancing overall patient comfort during the perioperative period.

## Materials and methods

Following approval from the Institutional Ethics Committee of SRM Medical College Hospital and Research Centre (1618/IEC/2019; SRM MCH RC), a randomized controlled trial was conducted in the department of anesthesiology in South India over a one-year duration. The trial has been registered under CTRI/2019/11/021855. The study focused on patients aged 40 to 65, falling within ASA grades 1 and 2, who were posted for inguinal hernia surgery under regional anesthesia. The study excluded individuals with a history of gastroesophageal reflux, hypothyroidism, and prior upper GI surgeries, as well as those with conditions like hemiplegia, and neurological disorders such as dystonia, chorea, and Parkinsonism. The study also excluded pregnant women, individuals with a BMI surpassing 35 kg/m2, and those taking medications that affect gastric motility. While diabetic patients were included, type 1 diabetics and type 2 diabetics on insulin and patients exhibiting symptoms of gastropathy or autonomic dysfunction were excluded.

Diabetic and non-diabetic patients were independently randomized into carbohydrate and non-carbohydrate loading subgroups using computer-generated random numbers. This approach resulted in the establishment of four distinct study groups, namely diabetic control, diabetic study, non-diabetic control, and non-diabetic study. The allocation of patients to their respective groups was accomplished using the sealed envelope technique.

Special attention was given to scheduling all study patients as the first case of the day, ensuring that they had their last solid meal the night before. In accordance with hospital protocol, patients received oral ranitidine and metoclopramide on the morning of their surgery. At 6 am, fasting blood sugar levels were measured. Patients in both the diabetic and non-diabetic "study" groups were provided with a 400-ml drink containing 50 g of dextrose, following recommended and established guidelines [7]. Patients in the diabetic and non-diabetic "control" groups received 400 ml of plain water.

**Figure 1:**
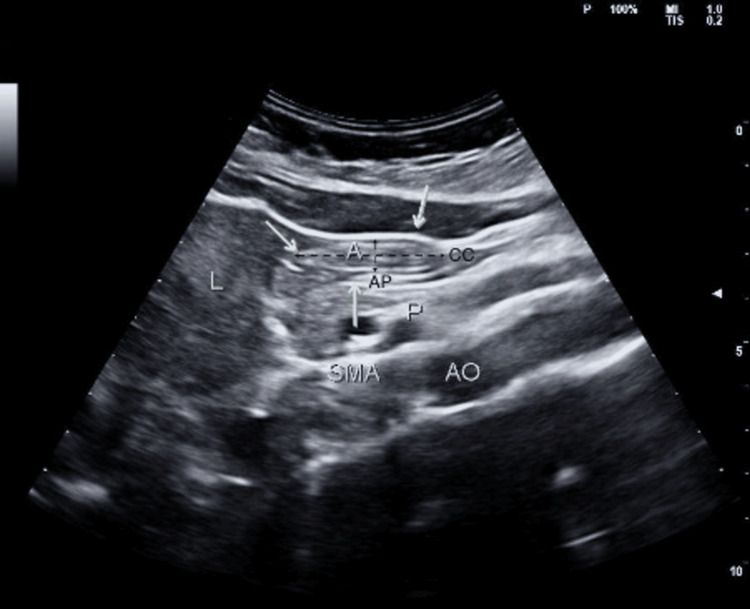
USG image of the gastric antrum A: antrum, AP: anterior-posterior diameter, CC: cranio-caudal diameter, L: left lobe of the liver, SMA: superior mesenteric artery, AO: aorta, P: pancreas

After two hours, once patients were transferred to the operating room, a USG examination of the stomach was performed for qualitative and quantitative estimation of gastric volume. This examination was carried out by a single trained anesthesiologist using a convex curvilinear probe (2-5 MHz) and the Philips ClearVue 350 USG machine from Koninklijke Philips, Netherlands. Patients were initially examined in the supine position and then in the right lateral decubitus (RLD) position for qualitative analysis. Quantitative measurements were taken while the patients remained in the RLD position (Figure 1). A cutoff value of gastric content volume less than 1.5 ml/kg was used as the criterion to proceed with anesthesia [8]. The thirst and discomfort levels of patients were assessed using a five-point Likert scale.

Capillary blood glucose (CBG) values were checked and patients with a post-dextrose loading CBG level exceeding 150 mg/dL were initiated on insulin treatment in accordance with the Vellore regimen and subsequently transferred to the operation room [9]. Once in the operating room, patients were continuously monitored for ECG, SpO2, and blood pressure. The administration of spinal anesthesia was carried out with stringent aseptic measures in place. Intraoperative CBG levels were monitored at hourly intervals following the commencement of surgery. Insulin as per the Vellore regimen was continued in patients whose CBG levels remained above 150 mg%. Normal saline 0.9% was administered based on the "4-2-1" rule to provide maintenance fluid. Subsequent to the surgical procedure, patients were transferred to the post-anesthesia care unit for postoperative monitoring.

Qualitative analysis of the gastric antrum

The assessment of the antrum involved specific criteria: it was deemed empty when the USG image displayed collapsed anterior and posterior walls. If the image depicted a cavity containing hyperechoic content with distended walls, it was categorized as containing liquid. Furthermore, if the image exhibited a "frosted glass" appearance resembling liver parenchyma, it indicated the presence of solid content [10]. Patients were subsequently classified according to this antrum analysis, and the classification details are provided in Table 1.

**Table 1:** Grading according to USG examination findings USG: ultrasound, RLD: right lateral decubitus

Grade	Significance of USG finding
Grade 0	Empty antrum in both supine and RLD positions, suggesting an empty stomach
Grade 1	The presence of liquid is apparent only in RLD suggesting a small amount of fluid in the stomach
Grade 2	The presence of liquid contents in both supine and RLD positions suggests the presence of increased gastric volume

Quantitative analysis of the gastric antrum

For quantitative analysis, the antral cross-sectional area (ACSA) was measured in the RLD position using two diameters of the cranio-caudal (CC) and antero-posterior (AP) of the antrum from serosa to serosa perpendicular to each other. Once the diameters were measured, using the Eclipse formula, we calculated the ACSA. ACSA = ((CC × AP) × 3.14)/4). After ACSA calculation, the stomach total volume (‘‘expected volume’’) was estimated for each subject using a previously tested and validated mathematical model. Stomach volume (ml) = 27 + 14.6 ACSA (cm2) − 1.28 age (years) [8,10].

Statistical analysis

**Figure 2:**
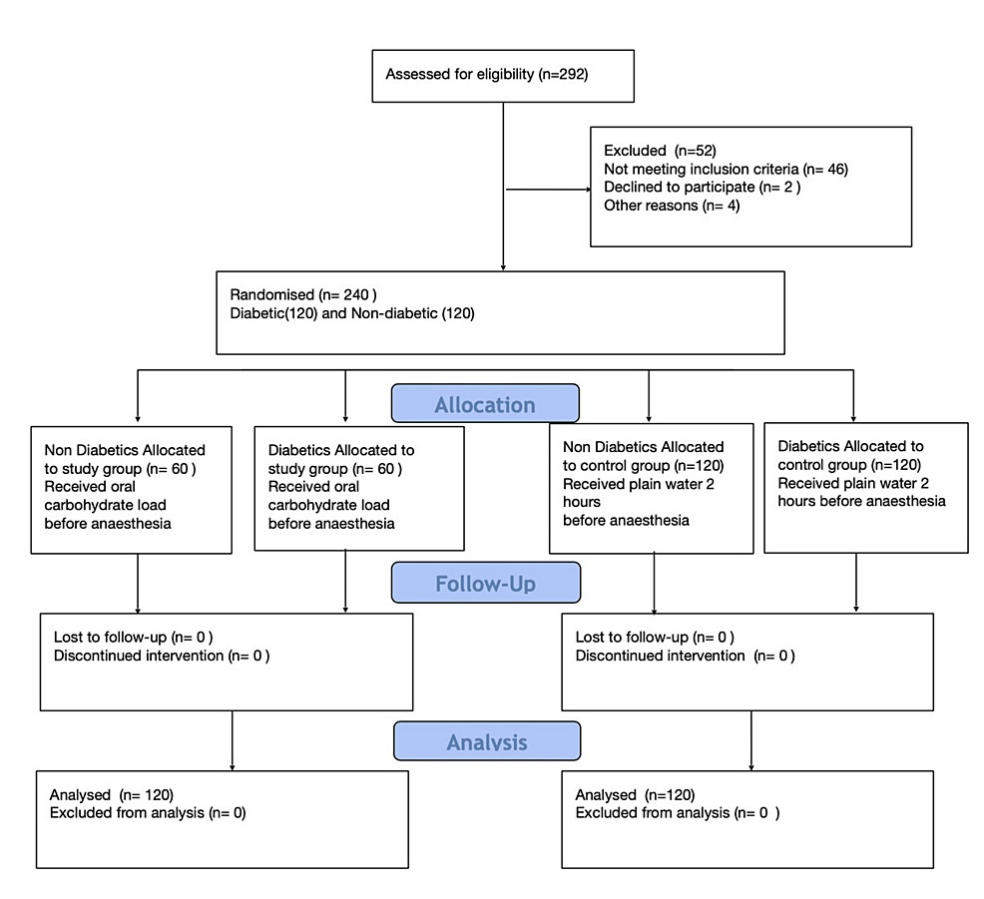
Consort diagram

Taking into account the diabetes prevalence of 14% in the general population and the total population of Tamil Nadu, India, which is 721,470,303, we initially calculated the sample size with a 95% confidence interval to be 186. Factoring in a 10% anticipated attrition rate, the final calculated sample size was 205. Ultimately, a total of 240 patients were enrolled, evenly divided into two groups, with 120 type 2 diabetic patients and the other 120 non-diabetic patients (Figure 2).

Statistical analysis was performed using SPSS Statistics version 25 (IBM Corp. Released 2017. IBM SPSS Statistics for Windows, Version 25.0. Armonk, NY: IBM Corp.). The data were normally distributed. An independent student t-test was done for quantitative data and a chi-square test was used for qualitative data across two groups. A comparison across four groups was made with a one-way ANOVA. A value of p<0.05 was considered to be statistically significant.

## Results

The demographic values of all the groups were comparable (Table 2).

**Table 2:** Demographic variables DC: diabetic control, DS: diabetic study, NDC: nondiabetic control, NDS: nondiabetic study, BMI: body mass index, ASA: American Society of Anaesthesiologist

Demographic	DC	DS	NDC	NDS	p-value
Age (years)	46±10.19	47.7±9.5	41.3±12.9	37.3±9.3	0.052
BMI (kg/m)	26.3±3.25	27.3±2.6	26.5±2.2	27.1±2.05	0.462
Gender	n (%)	n (%)	n(%)	n (%)	0.499
Male - number (%)	42 (70.0%)	40 (66.7%)	42 (70.0%)	48 (80.0%)	
Female - number (%)	18 (30.0%)	20 (33.3%)	18 (30.0%)	12 (20.0%)	
ASA I - number (%)	0 (0%)	0 (0%)	27 (45%)	25 (41.7%)	0.469
ASA II - number (%)	60 (100%)	60 (100%)	33 (55%)	35 (58.3%)	0.716

The duration of surgery ranged from 70 to 160 minutes (average: 109.99 minutes). All patients in the diabetic group belonged to ASA II, while the non-diabetic control group had 45% ASA I and 55% ASA II patients, and the non-diabetic study group had 41.7% ASA I and 58.3% ASA II patients.

Qualitative analysis of gastric volume in diabetic patients who had taken carbohydrate load (DS group) showed grade 0 in 39 patients (65%), grade 1 in 20 patients (33.3%), and grade 2 in one patient (1.66%). Diabetic patients who had not taken carbohydrate load showed grade 0 in 44 patients (73.3%) and grade 1 in 16 patients (26.7%) on qualitative analysis. There was no significant difference in USG grades between the control and study groups (p>0.05; Table 3). Qualitative analysis of gastric volume in non-diabetic patients who had taken carbohydrate load (DS group) showed grade 0 in 44 patients (73.3%) and grade 1 in 16 patients (26.7%). Non-diabetic patients who had not taken carbohydrate load also showed grade 0 in 44 (73.3%) and grade 1 in 16 (26.7%) on qualitative analysis. There was no significant difference in USG grades between the control and study groups in the nondiabetic population (p>0.05, Table 3).

**Table 3:** Assessment of gastric volume USG: ultrasound, DC: diabetic control, DS: diabetic study, NDC: nondiabetic control, NDS: nondiabetic study

USG grading	DC	DS	p-value	NDC	NDS	p-value	p-value across the group
Grade 0 - number (%)	44 (73.3%)	39 (65%)	0.262	44 (73.3%)	44 (73.3%)	1	
Grade 1 - number (%)	16 (26.7%)	20 (33.3%)		16 (26.6%)	16 (26.6%)		
Grade 2 - number (%)	0 (0%)	1 (1.67%)		0 (0%)	0 (0%)		
Gastric volume (ml)	35.3±12.95	31.2±11.75	0.163	29±11.42	30.4±9.12	0.518	0.182

Quantitative analysis of gastric volume did not show any significant difference in the diabetic population, with patients in the diabetic control group showing 35.3±12.95 ml and the diabetic study group showing 31.2±11.75 ml of gastric volume (p>0.05). One patient in the diabetic study group who had shown grade 2 in the qualitative analysis had a gastric volume of 95 ml which was above the cutoff range of 90 ml (1.5x body weight in kg). This case was postponed as the patient carried a high risk for aspiration. The gastric volume (ml) in the non-diabetic group also did not show any significant difference, with the NDC group having 29±11.42 ml and the NDS group having 30.4±9.12 ml of gastric volume (p>0.05).

Significantly higher CBG was noted in the second hour as compared to fasting CBG in the diabetic study group (143.9±24.9 and 183.2±28.67 mg/dL; p<0.05). Forty-five patients (75%) in the diabetic study group had a CBG value of more than 150 mg/dL, 15 patients (25%) were in the 150-199 mg/dL range, and 30 (50%) patients in 200-249 mg/dL range. Insulin infusion was started for these patients as per the Vellore regimen. In the non-diabetic study and control group, no significant difference in CBG values was noted (Table 4).

**Table 4:** Capillary blood sugar in the diabetic and non-diabetic population DC: diabetic control, DS: diabetic study, NCD: nondiabetic control, NDS: nondiabetic study, CBG: capillary blood glucose

DC	mg/dL	p-value	DS	mg/dL	p-value
Pre CBG	139.4±18.34	0.714	Pre CBG	143.9±24.9	0.000
Post CBG (2^nd^ h value)	138.6±15.8		Post CBG (2^nd^ h value)	183.2±28.67	
Post CBG (6^th^ h value)	138±17.22		Post CBG (6^th^ h value)	134.7±16.4	
NDC			NDS		
Pre CBG	95.5±8.52	0.001	Pre CBG	97.1±5.89	0.160
Post CBG (2^nd^ h value)	91.2±7.50		Post CBG (2^nd^ h value)	95.2±6.05	
Post CBG (6^th^h value)	93.7±6.76		Post CBG (6^th^h value)	95.5±4.32	

In this study, we assessed discomfort and thirst levels using Likert scores, with 0 indicating the least and 5 indicating the highest intensity. The diabetic control group reported a discomfort score of 2.9±0.55, while the diabetic study group exhibited a significantly lower score of 2.4±0.62 (p<0.05). Similarly, the nondiabetic control group registered a discomfort score of 3±0.61, whereas the nondiabetic study group displayed a significantly lower score of 2.3±0.65 (p<0.05). With respect to thirst, the diabetic control group reported a score of 4.8±0.44, whereas the diabetic study group showed a significantly lower score of 4.0±0.79 (p<0.05). In comparison, the nondiabetic control group had a thirst score of 4.7±0.53, and the nondiabetic study group exhibited a significantly lower score of 3.7±0.72 (p<0.05) (Table 5).

**Table 5:** Likert distribution of discomfort and thirst score DC: diabetic control, DS: diabetic study, NDC: non-diabetic control, NDS: non-diabetic study

	Likert scale	DC	DS	p-value	NDC	NDS	p-value
Discomfort	1	0	4	0.008	0	6	0.001
	2	10	27		10	28	
	3	42	27		38	24	
	4	5	2		8	2	
	5	3	0		4	0	
Thirst	1	2	16	0.036	8	24	0.001
	2	22	24		12	26	
	3	22	18		22	10	
	4	12	2		14	0	
	5	2	0		4	0	

## Discussion

Aspiration of gastric acid contents is a serious complication encountered during anesthesia, associated with significant morbidity. The fasting guidelines permit patients to take clear liquid until two hours before surgery, but a consensus has not been reached on guidelines for diabetic patients. Diabetics have been known to have delayed gastric emptying due to gastroparesis, putting them at an increased risk of aspiration [4,11]. In general, studies on gastric emptying have given conflicting data making it difficult to give proper guidelines for diabetic patients. Due to fear of delayed gastric emptying, diabetic patients are fasted more, inducing metabolic stress and increased insulin resistance [11].

William et al. studied the effects of carbohydrate-rich clear fluid given two hours before surgery. He found that it helps to reduce metabolic stress and insulin resistance, improve patient well-being, and reduce gastric emptying time. It was also noticed that carbohydrate loading improves postoperative glycemic control by stimulating endogenous insulin secretion [12].

We found no statistically significant change in gastric volume in diabetics and non-diabetics, irrespective of whether the patient was given carbohydrate loading or clear water. Indirectly, this implies that there is no significant delay in gastric emptying of liquids in either the diabetic or non-diabetic groups. One patient in the diabetic group had a borderline gastric volume of 95 mL. On a detailed examination, we found him to have autonomic symptoms like bloating, flatulence, and nocturnal diarrhea. The case was postponed for two hours as his gastric volume was close to 1.5 mL/kg (1.5x64 kg), the upper limit for the risk of aspiration [8]. After two hours, a repeat USG scan was done, and the stomach volume had decreased to 62 mL and the patient was taken up for surgery.

A study on gastric emptying after carbohydrate loading in 25 patients with type 2 diabetes using the paracetamol absorption technique found a decrease in gastric volume and a shorter gastric emptying time in diabetics compared to nondiabetics [13]. This correlated with this study in which there was a slight decrease in gastric volume of diabetic patients with oral carbohydrate load compared to the group who were given clear water. It has also been noted that emptying of semi-solid or solid is affected more than liquids in diabetics [12]. This opens the possibility of using a carbohydrate-rich beverage in order to delimit the stress-related metabolic derangement induced by prolonged fasting and will also help in optimizing post-surgical recovery. A study by Garg et al. compared gastric volume using USG in 53 diabetic and 50 non-diabetic patients after carbohydrate loading which showed no statistically significant difference in qualitative USG grading in either of the groups [14]. However, quantitatively, they found a significant increase in the cross-section area (CSA) of the antrum and thus the calculated gastric volume was high in the diabetic population. This was attributed to the fact that the 53 diabetic patients included in their study had symptoms suggestive of gastropathy, like bloating and gastric fullness. In our study, we found no statistically significant difference in qualitative or qualitative evaluation of gastric volume. However, we had one patient with autonomic symptoms who showed grade 2 on qualitative assessment and high gastric volume on quantitative assessment, similar to the study by Gary et al. Darwiche et al. studied the gastric emptying rate in 14 insulin-dependent diabetics and 19 healthy volunteers using USG after ingestion of semi-solid meals [14]. They found that the diabetic population had a significantly higher value of the antral area 90 minutes postprandial. Comparing this study with other studies, we infer that the delay in gastric emptying is greater for semi-solids than liquids in diabetics.

An increase in CBG (mg/dL) levels in the diabetic study group after carbohydrate loading compared with the diabetic control group is expected and has been reported in many studies [15,16]. A study by Gustafson et al. on non-surgical patients showed that CBG levels in diabetic and non-diabetic patients who received carbohydrate load were 241 and 137 md/dL, respectively. Sugars were found to peak at 60 and 30 minutes, respectively, after the carbohydrate load and return to baseline within 180 and 120 minutes, respectively [13]. In our study, there was a significant increase in CBG level two hours after carbohydrate loading in the diabetic group compared to the diabetics who were given clear fluids. We found that 75% of the diabetic patients had a sugar level of more than 150 mg/dL. They were managed with insulin infusions per the Vellore regimen. The elevated CBG level returned to baseline within one hour with an insulin infusion and remained close to the baseline after that.

A study was performed on 50 adult patients undergoing elective thyroidectomy where the study group was given 400 mL of oral carbohydrate-rich fluid two hours before surgery [17]. They were assessed for well-being based on thirst, nausea, vomiting, mouth dryness, fatigue, anxiety, and sleep quality. They found no statistically significant difference in any of the parameters in either group. They concluded that there was no effect of carbohydrate loading on the patient's well-being, as most patients were sleeping at night and were taken up as the first case in the morning. Therefore, they did not develop any additional discomfort due to the preoperative fasting. They suggested that the improvement in well-being might be more apparent if the patient was not taken up as the first case or if the patient was undergoing major surgery. In another study, 252 patients undergoing abdominal surgery were randomly subjected to carbohydrate fluid, placebo, and overnight fasting [18]. The carbohydrate group showed no increase in gastric fluid volume or acidity in the preoperative period. Hunger and anxiety were significantly (p<0.05) lower in the carbohydrate group compared to the other two groups. They concluded that giving a preoperative carbohydrate fluid can significantly improve patient well-being without adversely affecting gastric volume and pH. Bopp et al. studied 123 patients, randomized into two groups, one fasted overnight and another was given 200 mL of carbohydrate solution two hours before surgery, and were assessed for preoperative and postoperative thirst and hunger with a questionnaire [19]. The study group had significantly lower thirst and hunger preoperatively.

Limitation

Estimating serum insulin levels in diabetics can help determine the influence of carbohydrate loading on insulin resistance and provides scope for further study. Our study included only diabetics on oral hypoglycemic agents. Patients on insulin were excluded. A multicentric study with subgroups of patients requiring insulin and having autonomic symptoms can be done to know the dynamics of gastric emptying with different liquids.

## Conclusions

There is no increase in gastric volume or delay in gastric emptying in diabetics who were given plain water and carbohydrate-rich drinks. The comfort levels in the carbohydrate-loaded group were also better. We noted a higher glucose level in diabetics who consumed the carbohydrate-rich drink. We conclude that an extended duration of fasting in diabetics for fear of delayed gastric emptying is not warranted. Diabetic patients also can be allowed to have liquids until two hours prior to surgery, but it is preferable to avoid carbohydrate-based drinks as they increase the sugar level, necessitating the need for insulin.
